# Two sides of one coin: massive hepatic necrosis and progenitor cell-mediated regeneration in acute liver failure

**DOI:** 10.3389/fphys.2015.00178

**Published:** 2015-06-16

**Authors:** Hong-Lei Weng, Xiaobo Cai, Xiaodong Yuan, Roman Liebe, Steven Dooley, Hai Li, Tai-Ling Wang

**Affiliations:** ^1^Department of Medicine II, Section Molecular Hepatology, Medical Faculty Mannheim, Heidelberg UniversityMannheim, Germany; ^2^Department of Medicine II, Saarland University HospitalHomburg, Germany; ^3^Department of Gastroenterology, Ren Ji Hospital, School of Medicine, Shanghai Jiao Tong UniversityShanghai, China; ^4^Department of Pathology, Beijing China-Japan Friendship HospitalBeijing, China

**Keywords:** massive hepatic necrosis, acute liver failure, acute-on-chronic liver failure, liver progenitor cell, liver regeneration

## Abstract

Massive hepatic necrosis is a key event underlying acute liver failure, a serious clinical syndrome with high mortality. Massive hepatic necrosis in acute liver failure has unique pathophysiological characteristics including extremely rapid parenchymal cell death and removal. On the other hand, massive necrosis rapidly induces the activation of liver progenitor cells, the so-called “second pathway of liver regeneration.” The final clinical outcome of acute liver failure depends on whether liver progenitor cell-mediated regeneration can efficiently restore parenchymal mass and function within a short time. This review summarizes the current knowledge regarding massive hepatic necrosis and liver progenitor cell-mediated regeneration in patients with acute liver failure, the two sides of one coin.

Massive hepatic necrosis (MHN) denotes an extensive and diffuse necrosis spanning multiple lobes or multiple acini in healthy or diseased liver. MHN has many synonymous terms, for example, massive hepatic loss, acute yellow atrophy, fulminant massive necrosis, panacinar necrosis, confluent necrosis, multilobular necrosis et al. (Kalk, 1959; Horney and Galambos, 1977; Craig et al., 2004). Clinically, MHN is a critical event underlying acute liver failure (ALF), a serious clinical syndrome associated with a high mortality (Sugawara et al., 2012). Besides ALF, MHN also occurs in chronically diseased livers suffering from acute functional decompensation (Popper and Elias, 1955), a recently recognized disease entity entitled acute-on-chronic liver failure (ACLF). Livers of the patients recovered from ALF or ACLF demonstrate a remarkable regenerative capacity to rapidly restore lost parenchymal cells and corresponding functions. This pattern of liver regeneration is thought to be mediated by liver progenitor cells (LPC) (Desmet, 2011a,b) and entitled the “second pathway of liver regeneration” to be distinguished from the “first pathway of liver regeneration,” in which repopulation of the liver is dependent on the remaining mature hepatocytes (Clouston et al., 2009). Necrosis and LPC-mediated regeneration comprise two sides of one coin, the balance of which determines the destiny of patients suffering from MHN.

This review discusses defining pathological features of MHN as well as the role and potential mechanisms of LPC-mediated liver regeneration in patients with liver failure. We mainly focus on the knowledge gained from human studies. Table 1 summarizes the main references supporting the views expressed in this review.

**Table 1:** **Selected clinical references investigating massive hepatic necrosis and liver progenitor cell in acute or acute-on-chronic liver failure**.

**Authors and Time**	**Findings**	**Comments**	**References**
Lucke, B. (1944) and Lucke, B. and Mallory, T. (1946)	The two studies based on 296 dead patients with autopsy described detailed pathological features and clinicopathologic correlation of fatal hepatitis (virus-induced ALF).	The most complete and thorough investigations into MHN and pathological features of ALF so far. The studies not only uncovered the basic pathological features of MHN during ALF, but also pointed out the huge difficulty in clinical investigation of MHN: clinicians have few chances to obtain liver samples where MHN is occurring.	Lucke, 1944; Lucke and Mallory, 1946
Popper, H. and Elias, H. (1955)	This study proposed that the process is similar whether massive necrosis occurs in a previously healthy person or in a patient with cirrhosis.	This study performed 60 years ago suggested that ACLF has the similar pathological features as ALF.	Popper and Elias, 1955
Boyer, J. L. and Klatskin, G. (1970)	This study showed that liver biopsy can successfully discriminate patients with “subacute hepatic necrosis” from those with “classic necrosis” according to presence or absence of pan-lobular necrosis.	Liver biopsy is a good tool to monitor MHN.	Boyer and Klatskin, 1970
Hanau, C., et al. (1995)	This study showed histopathological heterogeneity in fulminant hepatic failure. In addition, the authors proved that the percentage and distribution of necrosis was not associated with clinical outcome.	The study reminds clinicians of the potential misleading results of percutaneous liver biopsy due to regional inhomogeneities.	Hanau et al., 1995
Chenard-Neu, M. P., et al. (1996)	This study showed that the percentage and distribution of necrosis at the time of transplantation was not associated with the final outcome.	The two studies (Hanau's and Chenard-Neu's) suggest that the extent of MHN should not be considered a prognostic marker of ALF.	Chenard-Neu et al., 1996
Katoonizadeh, A., et al. (2006)	This study proposed that 50% loss of hepatocytes is a threshold for extensive activation of LPCs due to a significant decrease of proliferative activity in the remaining hepatocytes.	This impressive clinical study suggests a potential link between severe hepatocyte death and activation of LPC.	Katoonizadeh et al., 2006
Liu, Q, et al. (2007)	This study showed that the newly formed hepatocytes are not yet effectively connected to the biliary system due to marked ischemia and obstruction of intralobular canaliculi.	The study points out a key issue in ALF: Connection between the newly formed hepatocytes and existing biliary tree determines whether these hepatocytes have complete functions.	Liu et al., 2007
Farci, P., et al. (2010)	This study showed that overwhelming response of humoral immunity may play a role in MHN.	The study explores an important role of humoral immunity in MHN.	Farci et al., 2010
Dechene, A. et al. (2010)	This study showed a positive correlation between parenchymal loss, LPC proliferation and HSC activation.	The study suggests potential interactions between LPC proliferation and activated HSCs during liver regeneration after MHN.	Dechêne et al., 2010
Stravitz, R. T, et al. (2011)	This study proposed a novel category of MHN based on special characters of autoimmune hepatitis.	This new category of MHN suggested an important impact of etiology on the pathogenesis of MHN.	Stravitz et al., 2011
Nissim, O., et al. (2012)	This study investigated expression gene profiles in 2 HBV patients who underwent MHN.	The study provides important information about global changes of gene expression in liver tissues after MHN.	Nissim et al., 2012
Dechene, A., et al. (2014)	This study showed that performing liver biopsy using mini laparoscopy in patients with ALF and severe coagulopathy was safe. Detecting Ki67 and M30 with IHC staining may identify patients who would recover spontaneously or who would need a liver transplant.	The study provides further evidence suggesting that liver biopsy is a very useful tool in monitoring disease progression in ALF.	Dechêne et al., 2014
Li, H., et al. (2015)	This study confirmed that massive hepatic necrosis is the pathological feature of ACLF, at least in cirrhotic patients with HBV infection. This study also showed that extent of necrosis did not correlate with clinical outcome.	The study confirms Popper's view that the process is similar whether massive necrosis occurs in a previously healthy person or in a patient with cirrhosis.	Li et al., 2015

## Pathological characteristics of MHN

**Figure 1:**
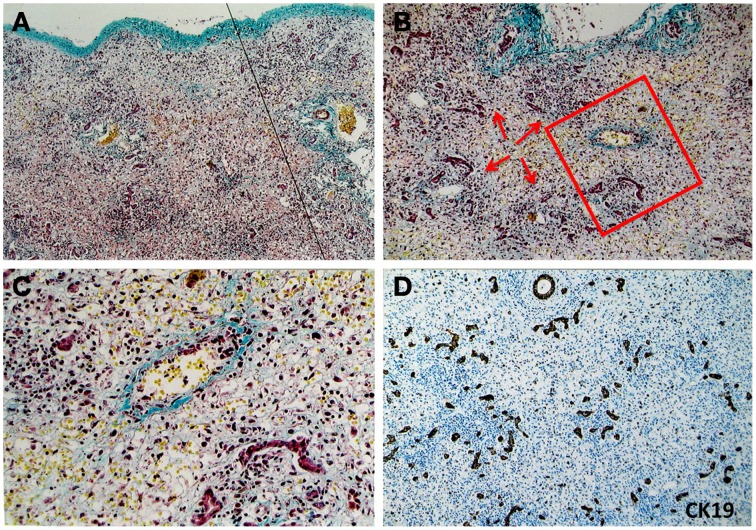
**Pathological feature of massive hepatic necrosis**. A representative liver tissue was obtained from a patient with acute HBV infection who had undergone liver failure for 4 days. **(A)** A line depicts that the liver parenchyma had been completely destroyed. Masson staining highlights shrinkage of the liver capsule (green color in upper limit of the tissue). **(B–C)** Remarkable inflammatory cells (arrows) infiltration into destructive areas with ductular reaction is shown. Endophlebitis of the terminal veins is highlighted by a frame and magnified in **(C)** Masson staining. **(D)** Ductular reaction is further confirmed at the necrotic liver using immunohistochemical staining for CK19.

Seventy years ago, two elegant studies investigated detailed pathological features of MHN (Lucke, 1944; Lucke and Mallory, 1946). In these studies, Lucke and colleagues collected a wealth of clinical data from autopsied specimens of different organs of patients who died in an outbreak of “fatal hepatitis” in the Army of the United States between 1942 and 1945. The two cohorts in this series comprised of patients whose death and autopsy time occurred either less or more than 10 days after onset of the disease, respectively. Patients with MHN displayed the following histological features:

1. In patients with a clinical course of less than 10 days, the lesion involved all parts of the liver uniformly. In many cases, tissue destruction was extreme and complete. But it was also frequently observed that a few hepatocytes persisted at the lobular periphery, forming a narrow rim. In patients with a clinical history of more than 10 days, destruction of the liver was less uniform. The parenchyma was completely obliterated in large areas, whereas destruction was incomplete elsewhere.
2. The destruction extended from terminal veins to periphery of lobes.
3. Dead cells were removed very rapidly. The earliest stages of cell disintegration could not be observed. Even in the most rapidly succumbing patients, no traces of dead cells could be found.
4. The destruction specifically affected hepatocytes, whereas the framework and sinusoids remained unaltered.
5. No scar occurred in necrotic areas.
6. Remarkable inflammatory reaction accompanied the destructive process. Inflammatory cellular infiltration was considerably more marked in the acute stage (<10 days) than that in the subacute form (>10 days). Macrophages/monocytes were the most prominent infiltrating cells. The most conspicuous inflammation was localized at the lobular periphery.
7. LPC-mediated ductular reaction (DR) and regeneration occurred at an extremely early phase of destruction and persisted for a long time.
8. In patients with a clinical history of more than 10 days, endophlebitis of the efferent veins was conspicuous, whereas the phenomenon was less common in patients with a clinical history of less than 10 days. In these early cases, the walls of the terminal veins were substantially thickened and of homogenous, hyaline texture. In the latter group, fibrous obliteration of the smaller veins was more common than inflammatory reaction of the vessels.
9. Jaundice and different forms of cholestasis were principal features in MHN patients with a long-term clinical history. However, jaundice was less common in patients with a clinical history of less than 10 days. Figure 1 shows an HBV patient who had undergone acute liver failure for 4 days. The liver parenchyma has been completely obliterated (Figure 1A). Remarkable inflammatory cell infiltration into destructive areas (Figure 1B) with endophlebitis of the terminal veins (Figure 1C) is observed. Strong ductular reaction can be detected at this moment (Figure 1D).

These two seminal studies, which included 296 dead patients with autopsy (most of whom were USA soldiers), represent the most complete and thorough investigations into MHN so far. In times of peace, studies like these, based on multiple autopsy samples, are no longer feasible. Thus, the findings from these two studies remain unique and relevant even 70 years later.

Besides pathological features of MHN, Lucke's studies also provided impressive data on clinicopathological correlations. One of the pivotal findings is the non-correlation between liver lesion and clinical duration. No relationship can be established between duration of the disease, inflammatory reaction and the degree of liver regeneration (Lucke, 1944; Lucke and Mallory, 1946). Marked regeneration may be present in some patients with a short disease course, but absent in others with a long clinical history. Similar phenomena are also observed in patients with ACLF (Li et al., 2015).

A key issue that Lucke did not answer is whether the extent of necrosis has a direct impact on the final clinical outcome of patients, e.g., incidence of multiple organ failure and death. Subsequent studies from Hanau's and Chenard-Neu's groups address this question (Hanau et al., 1995; Chenard-Neu et al., 1996). In Hanau's study, half of the enrolled patients had MHN (defined as parenchyma loss >90% of the entire liver), whereas others had up to 70–90% preservation of hepatocytes. However, survival rate was low in all patients, regardless of parenchymal preservation. In addition, the study did not find a correlation between the extent of necrosis and the development of intracranial hypertension, the most common cause of death in ALF (Hanau et al., 1995). The findings were further supported by Chenard-Neu's work. In a clinical study to investigate the role of auxiliary liver transplantation in treating 30 patients with ALF, Chenard-Neu and colleagues showed that the percentage and distribution of necrosis at the time of transplantation was not associated with the final outcome (Chenard-Neu et al., 1996).

In addition to patients with ALF, who have healthy livers before disease onset, MHN also occurs in patients with cirrhosis (Popper and Elias, 1955). In a recent clinical study, we investigated 174 transplanted livers with HBV-related cirrhosis (Li et al., 2015). Among them, 69 had severe necrosis between 15–90% of the entire liver. We separated patients with serious necrosis into two groups according to area of necrosis (15–33% or 33–90%) and compared critical clinical parameters, including creatinine, serum total bilirubin, international normalized ratio (INR), disease severity scores [model for end-stage liver disease (MELD), Child–Pugh, and acute physiology score, age points, chronic health points (APACHE) III] and organ-failure scores [chronic liver failure-sequential organ failure assessment score (CLIF-SOFA), sequential organ failure assessment score (SOFA)] at different time points before liver transplantation between both groups. Patients with small (15–33%) or large (33–90%) areas of necrosis had an identical clinical outcome. These results suggest that occurrence of MHN, i.e., the event itself, rather than the extent of necrosis, is critical in the progression of liver failure and extrahepatic organ failure in patients with ALF or ACLF.

## Massive hepatic necrosis: definition and classification

So far, a consensus definition of MHN has not yet been agreed upon. Massive hepatic necrosis is a morphological concept. The core characteristic of MHN is extensive multilobular/panacinar hepatocyte necrosis (Craig et al., 2004). MHN was previously defined according to the extent of necrosis. For example, MHN was defined by some experts as extensive, diffuse panlobular (panacinar) and multilobular necrosis of >60–70% as noted on examination of the entire liver on explant, autopsy, or clinical visualization (Alastair et al., 2012). The term submassive hepatic necrosis (SMHN) has sometimes been used for lesions that involve global necrosis of 30–70% of the entire liver (Alastair et al., 2012). In addition, Hanau and colleagues categorized MHN when a whole liver showed nearly 100% necrosis, whereas SMHN was termed as a liver with necrosis of between 15 and 90% (Hanau et al., 1995). These definitions based on the extent of necrosis are artificial. Except in autopsied or transplanted livers, calculating necrotic percentage of the entire liver is impossible in routine clinical practice. The key question is whether the extent of hepatocyte necrosis affects the clinical outcome of patients. As mentioned above, the percentage and distribution of necrosis at the time of liver examination is not correlated with the final clinical outcome of patients with ALF. Thus, a rational definition of MHN is still at large. Clinical markers and biomarkers, which reflect patho-physiological change in the damaged liver, e.g., MHN and regeneration, or clinical outcome of patients undergoing ALF are missing.

Recently, an acute liver failure study group proposed a novel category in autoimmune acute liver failure by classifying several variants of MHN as MHN1–MHN5 (Stravitz et al., 2011). MHN1 and MHN2 are equivalent to classic MHN and SMHN. Bridging hepatic necrosis or multilobular necrosis with neocholangiolar proliferation was defined as MHN3. MHN4 and MHN5 were defined as serious necrosis combined with features of autoimmune hepatitis. This classification suggests an important impact of etiology on the pathogenesis of MHN.

## Mechanism of MHN

The mechanisms underlying MHN are largely unknown so far. As mentioned above, massive hepatic necrosis is a traditional morphological concept in this review. Thus, the term “necrosis” does not denote that necrosis, a mode of cell death often juxtaposed to apoptosis, is the major mechanism underlying MHN. The disease progression in patients with MHN is dramatic. The timespan between the initiation of symptoms and death can be as little as 1 day (Lucke and Mallory, 1946). On the other hand, the dead cells in necrotic livers are removed very rapidly (Lucke and Mallory, 1946). These features make investigations into the mechanisms of MHN very difficult. Thus, the relative contribution of apoptosis or necrosis to MHN in patients is controversial (Schulze-Osthoff and Bantel, 2011). So far, most investigations to delineate the mechanism have been performed in animal models (comprehensively reviewed by Bantel and Schulze-Osthoff, 2012). Clinically, elevated levels of death ligands or receptors, e.g., CD95L, CD95, TRAIL, TNF-α, TNF receptors and Caspases, have been reported in ALF patients induced by different etiologies (Strand et al., 1998; Ryo et al., 2000; Streetz et al., 2000; Tokushige et al., 2000; Nakae et al., 2001; Rivero et al., 2002; Volkmann et al., 2008), suggesting an association between apoptosis and MHN in progression of ALF. On the other hand, necrosis is considered a prominent death pathway of hepatocytes in some drug-induced ALFs. For example, acetaminophen metabolism leads to generation of toxic metabolite N-acetyl-p-benzoquinone that induces loss of mitochondrial membrane potential and depletion of ATP in hepatocytes (Hinson et al., 2010).

More recently, necroptosis has been reported to play a critical role in cell death during liver disease and ALF (Luedde et al., 2014). Necroptosis is a newly discovered Caspase-8 regulated necrotic pathway, which requires receptor interacting protein kinase 3 (RIP3) and phosphorylated Mixed Lineage Kinase Domain-like Protein (MLKL) (Pasparakis and Vandenabeele, 2015). Wang and colleagues found significantly elevated levels of phospho-MLKL in liver tissues from 14 patients with drug induced liver injury (DILI) compared to normal liver specimens (Wang et al., 2014). In the acetaminophen-induced mouse ALF model, inhibition of RIP3 protein or the use of RIP3-deficient mice strongly decreased necrotic cell death in the early phase of liver damage, however, the protective effects was lost after 24 h (Ramachandran et al., 2013). Further, investigations are required to elucidate the role of necroptosis in ALF and ACLF.

It should be emphasized that most of the clinical studies to date measured the levels of death ligands/receptors only in blood samples. Even if some measurements were performed in liver tissues, the time point investigators collected the samples might be not the time hepatocytes were dying. Thus, which pathways, apoptosis, necrosis or necroptosis, and how these pathways contribute to MHN remains unclear. Very likely, etiology and duration of the disease determine the nature and extent of cell death in MHN.

Recently, Farci and colleagues investigated explanted liver tissues from two patients with HBV-associated ALF and found a massive accumulation of plasma cells secreting IgG and IgM and complement deposition within necrotic areas. These results suggest an overwhelming response of humoral immunity in ALF (Farci et al., 2010). In addition, they found that the molecular target of these antibodies is the hepatitis B core antigen (HBcAg) (Farci et al., 2010). However, how and to what extent the B cell response contributes to massive necrosis in HBV-associated ALF needs further investigation.

Besides the mechanisms underlying MHN, a lot of other interesting questions have not been answered yet. For instance, why is it only hepatocytes that die during MHN? Why does the sinusoidal framework remain intact during MHN?

## Liver progenitor cells mediate liver regeneration in MHN

It is not clear why some individuals recover from ALF spontaneously while others die. Although parenchymal cells are almost completely lost in the process of MHN, the liver still demonstrates an enormous regenerative capability to restore liver parenchyma and function, which is thought to be dependent on liver progenitor cells residing in the Canals of Hering and the ductules (Gouw et al., 2011; Desmet, 2011a,b; Itoh and Miyajima, 2014). The final clinical outcome is dependent on whether the massive parenchymal loss can be rapidly compensated by LPC-mediated regeneration. In Lucke's studies, “proliferative changes in bile ducts” accompanied massive necrosis in the patients with acute liver failure. They described that “in most cases, even the most acute, the small twigs of the bile ducts, both septal (perilobular) and interlobular, exhibit some evidence of proliferation. These little ducts normally are inconspicuous.” The “proliferative changes in bile ducts” presented as “the ducts have irregular shapes, due to budding and branching, and the nuclei of the component cells are deeply chromatic.” Interestingly, in some of patients the clinical duration was less than 1 day (Lucke and Mallory, 1946). These “proliferative changes in bile ducts” can be attributed to activation and proliferation of LPC-mediated ductular reaction.

Ductular reaction is defined as a reaction of ductular phenotype, possibly but not necessarily of ductular origin, in acute and chronic liver disease (Roskams et al., 2004). DR may arise from different cell sources in different settings, including (1) proliferation of pre-existing cholangiocytes; (2) liver progenitor cells (local and/or circulating cells probably bone marrow-derived); (3) rarely, biliary metaplasia of hepatocytes; (4) hepatocytes (Desmet, 2011b). In MHN, LPCs are the predominant cell source of DR because most parenchymal cells die in this setting. In chronic liver disease with different etiologies (for example, NASH, ASH, HBV, HCV, and hemochromatosis), the degree of DR correlates with disease severity, e.g., inflammation and fibrosis (Roskams et al., 2003; Clouston et al., 2005; Richardson et al., 2007; Weng et al., 2013; Gadd et al., 2014; Wood et al., 2014). In these settings, occurrence of DR is closely associated with chronic liver injury-induced hepatocyte senescence and proliferative arrest of hepatocytes. In contrast to mature hepatocyte derived “primary hepatic regeneration” (Clouston et al., 2005; Richardson et al., 2007), DR-related regeneration was considered the “secondary pathway of liver regeneration,” which is regulated by hepatic stellate cells, inflammatory cells such as macrophages and extracellular matrix (Clouston et al., 2009). Multiple cytokines (IL-6 Matthews et al., 2004, TNF-α/TWEAK Jakubowski et al., 2005, TGF-β Nguyen et al., 2007, and IFN-γ Knight et al., 2007; Weng et al., 2013), growth factors (HGF and EGF) (Campbell et al., 2001), hormones (insulin and somatostatin, Jung et al., 2010, 2012), neurotransmitters (Cassiman et al., 2002), transcription factors (NFκB Tirnitz-Parker et al., 2010), and signaling pathways (Jak/STAT Yeoh et al., 2007, Wnt, Spee et al., 2010; Boulter et al., 2012, Notch, Spee et al., 2010; Boulter et al., 2012; Sicklick et al., 2006; Omenetti and Diehl, 2008; Omenetti et al., 2008, 2009) have been recognized to regulate the process. Detailed descriptions are provided in various reviews elsewhere (Gouw et al., 2011; Best et al., 2013; Williams et al., 2014).

In contrast to chronic liver diseases, the regenerative process that follows MHN has not yet been intensively investigated. Several studies described robust DR in patients with ALF (Katoonizadeh et al., 2006, 2010; Dechêne et al., 2010; Rastogi et al., 2011). Katoonizadeh reported that 50% loss of hepatocytes is a threshold for extensive activation of LPCs due to significant decrease of proliferative activity in the remaining hepatocytes (Katoonizadeh et al., 2006). Given that liver failure occurs within a very short time, several factors may determine whether LPC-dependent liver regeneration can save the failing liver, including the number of activated cells, speed of cell proliferation, and direction of cell differentiation.

So far, it is not clear which proportion of LPCs is activated and how fast the LPC proliferation is proceeding in ALF. In chronic liver disease, some studies based on animal models reported that the activation and expansion of the LPCs requires more than 7 days, while LPC differentiation into intermediate hepatocyte requires another 7 days (Fausto, 2004; Fausto et al., 2006). Obviously, LPC expansion and differentiation at such low speed cannot rapidly restore hepatocyte mass and function due to MHN. Actually, in patients with MHN, “proliferative changes in bile ducts” occurred even within 1 day after initiation of disease (Lucke and Mallory, 1946), indicating that LPC mobilization during MHN happens at high speed and extent. Recently, work from Nissim and colleagues provided clues to the potential contribution of LPCs in ALF (Nissim et al., 2012). In this study, they investigated liver regeneration signature in 4 liver tissues (2 with MHN and 2 with SMHN) from patients with HBV-associated ALF and 10 donated livers from volunteers using microarray analyses (Nissim et al., 2012). Four patients underwent liver transplantation within 8 days from the onset of symptoms. Compared with control livers, all patients with ALF demonstrated highly up-regulated gene expression associated with LPC markers, including CK19 and CK7, EPCAM, CD133/PROM1, CD24, SOX9, THY1, CD44, NCAM1, and CK8. Among 4 livers with severe necrosis, 2 livers with MHN had much higher expression of LPC-associated genes than the other 2 with SMHN, suggesting that the MHN induces the highest expression of LPC-associated genes.

**Figure 2:**
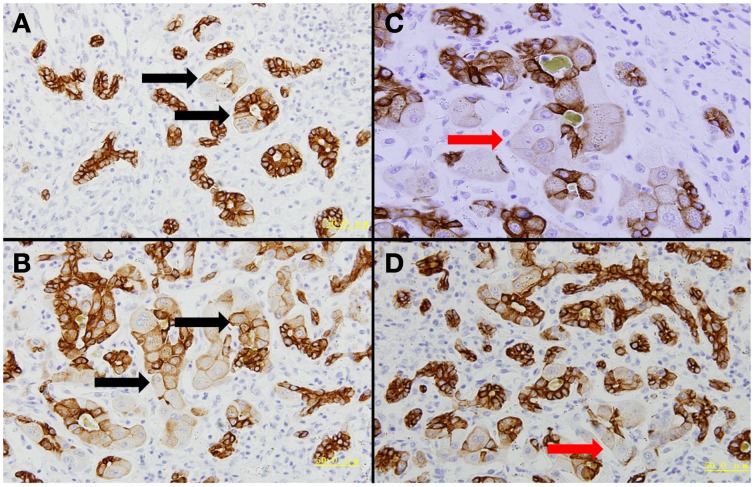
**Dual differentiation of liver progenitor cells**. The presented liver tissue was from a patient with acute HBV infection who had undergone liver failure for 13 days. CK7 immunohistochemical staining was performed. Four fields were selected to show that liver progenitor cells contribute to rebuild bile ducts and hepatocytes. **(A–B)** Liver progenitor cell-originated bile ducts still can be discriminated by hepatocyte-like cells inside these ducts (black arrows). **(C–D)** Liver progenitor cell-derived hepatocytes are highlighted by red arrows.

LPCs possess bi-potential capacity, being able to differentiate into either hepatocytes or cholangiocytes (Roskams, 2003, 2008). Figure 2 shows the typical dual differentiation of LPC in an HBV patient 13 days after liver failure. In the process of MHN, whether LPCs differentiate to hepatocytes with sufficient speed and in substantial numbers determines whether the damaged liver can restore parenchymal mass and function (Desmet, 2011a,b). In acute and chronic liver disease, the balance between Wnt and Notch signaling is considered a key mechanism to steer LPC differentiation in either direction (Spee et al., 2010; Boulter et al., 2012). Prominent Wnt signaling causes LPC differentiation into hepatocytes whereas Notch signaling drives LPC differentiation toward cholangiocytes. It is unknown whether these two signaling pathways play similar roles in MHN. Analyzing microarray data in ALF, Nissim found up-regulated genes associated with Wnt and Notch signaling pathways, however, key component of these pathways were not detected (Nissim et al., 2012).

Several factors may impact on LPC differentiation into hepatocytes in MHN, including inflammatory and fibrotic microenvironment. Distinct from other acute and chronic liver disease, acute liver failure occurring in either a previously normal liver or a cirrhotic liver has a unique immune response and inflammatory cell profile in the process of disease progression. Early in the course of ALF, severe injury of the liver recruits activated monocytes from circulation and bone marrow to enter the liver. These monocytes release high levels of pro- and anti-inflammatory cytokines simultaneously both systemic and in the liver that induces the systemic inflammatory response syndrome (SIRS) and compensation anti-inflammatory response syndrome (CARS) (Possamai et al., 2010). Subsequently, imbalance of the two opposing forces steers the immune response toward an anti-inflammatory milieu and causes functional monocyte deactivation, sepsis and even multiple organ dysfunction syndrome (MODS) (Wasmuth et al., 2005). So far, no data exist as to whether the big changes of inflammatory milieu impact DR after MHN. It seems that in most cases DR continues to develop with time after MHN regardless of the big changes of the inflammatory milieu.

In addition to the inflammatory environment, wound healing in damaged liver may significantly affect LPC-mediated liver regeneration (Kordes and Haussinger, 2013; Kordes et al., 2014). Wound healing does not equate to fibrosis although both are characterized by activation of local myofibroblasts (including hepatic stellate cells and portal fibroblasts) and subsequent deposition of extracellular matrix (ECM) in different organs (Wynn, 2008). Nissim's microarray analyses revealed that the extent of liver necrosis was correlated with a prominent fibrogenesis gene signature (Nissim et al., 2012). Dechene's study also showed a positive correlation between parenchymal loss, LPC proliferation and HSC activation (Dechêne et al., 2010). In chronic liver damage, the relationship between LPC proliferation and HSC activation has been intensively investigated. In an animal model feeding a choline-deficient, ethionine-supplemented (CDE) diet, Van Hul and co-workers found that HSC activation and collagen deposition occurred after 3 days of CDE feeding while LPC proliferation was manifested after 7 days. During the progression of liver damage, LPCs were embedded in ECM at all times (Van Hul et al., 2009). The phenotypic orientation of LPCs in this process was regulated by macrophages (Van Hul et al., 2011). However, the scenario unraveled in chronic liver disease obviously does not match that occurring in MHN. As mentioned above, activation and proliferation of LPCs in liver with MHN occurred rapidly (even within 1 day) (Lucke and Mallory, 1946). Under these circumstances it is not likely that HSC activation and ECM secretion are a prerequisite for further LPC activation. Thus, signaling events that switch activation of LPC in MHN may be dependent on parenchymal loss. In addition, HSC activation and collagen deposition in this setting are more likely a physiological repair response because there is no scar tissue (fibrosis) occurring in necrotic areas during MHN (Lucke, 1944). The role and mechanisms of inflammation and fibrosis in MHN should be intensively investigated in the future.

Whether the newly formed hepatocytes have full biological function depends on the connection between the canaliculi of new hepatocytes and the existing biliary tree. However, in most of cases the newly formed hepatocytes show ballooning degeneration due to marked ischemia and obstruction of intralobular canaliculi, suggesting that the newly formed functional hepatocytes are not yet effectively connected to the biliary system (Liu et al., 2007).

## Massive necrosis in acute-on-chronic liver failure

In addition to patients with ALF, who have healthy livers before the disease onset, MHN also occurs in patients with cirrhosis. In recent years, ACLF has been established as a distinct disease entity derived from patients with cirrhosis or non-cirrhosis with acute decompensation (Sarin et al., 2009; Canbay et al., 2011; Jalan et al., 2012, 2014; Moreau et al., 2013). The disease is characterized by several core clinical features, including acute deterioration of hepatic functions, multiple-organ failure and deranged systemic inflammatory responses (Sarin et al., 2009; Laleman et al., 2011; Olson and Kamath, 2011; Gines et al., 2012; Jalan et al., 2012). However, the pathological characteristics and underlying pathophysiology of ACLF have not been completely clarified so far (Jalan et al., 2014).

As mentioned above, we organized a prospective single-center study recently, in which 174 patients undergoing liver transplantation due to acute decompensation of hepatitis B virus (HBV)-associated liver cirrhosis were investigated (Li et al., 2015). In this study, MHN and SMHN were defined as necrosis of more than 90% and 15–90% of the entire liver on explant, respectively (Hanau et al., 1995). However, in this cohort of patients, none had necrotic areas exceeding 90%. SMHN was identified in 69 patients with cirrhosis. These patients demonstrated typical core clinical features of ACLF: acute deterioration of hepatic functions, multiple-organ failure and deranged systemic inflammatory responses. The results suggest that massive necrosis (defined as SMHN in this study) is a pathological feature of ACLF in cirrhotic patients with HBV infection.

In principle, the process is similar whether massive necrosis occurs in a previously healthy person or in a patient with cirrhosis (Popper and Elias, 1955). However, cirrhotic livers disrupted by interconnecting fibrous scars and distorted vessel systems also endow some morphological features distinct from healthy livers, although both suffer from acute decompensation and develop into liver failure. For example, in a cirrhotic liver with massive necrosis, necrotic areas of more than 90% of the entire liver are rare. Microscopically, various amounts of cirrhotic nodules were remaining in all livers with ACLF. Although detailed mechanisms underlying these phenomena are not clarified, several factors may be associated. As we know, massive necrosis extends from terminal vein (zone 3) to the portal tracts (zone 1) in both healthy livers (ALF) and in cirrhotic livers (ACLF). In ALF, the spread of parenchyma loss is unstoppable. The necrosis can extend to neighboring lobes and even to the entire liver within a very short time. However, this kind of complete necrosis diffusion observed in a previously healthy liver is impossible in a cirrhotic liver, where fibrogenesis and vascular system remodeling has isolated hepatic parenchyma into countless “islands” of different size (Alastair et al., 2012). Within fibrous septa, extensive collagen crosslinks develop, which are irreversible and not degraded by enzymes like collagenases (Brenner, 2013). These septa serve as “useful barriers” to prevent necrosis from spreading across the cirrhotic liver. In addition, remodeling vascular system may also impact the diffusion of necrosis in ACLF. In cirrhotic livers, fibrous septa that link central veins and portal veins/hepatic arteries establish anastomoses between two or more draining vessels, which alter normal hepatic vasculature (Desmet, 2011b). These fibrous vascularized septa linking central veins and portal tracts create “a bridge too far” (Desmet and Roskams, 2003). The direct anastomoses between afferent (hepatic artery and portal vein) and efferent (centrolobular veins) vessels force a fraction of the blood to bypass the lobular parenchyma without functionally contacting between blood and hepatocytes (Desmet, 2011b). These hepatic vascular alterations combined with frequently occurring hepatic and portal thromboses induce obliteration of small veins (e.g., terminal veins) and local parenchymal extinction in cirrhotic livers (Wanless et al., 1995, 2000). Microscopically, small regions of extinction are easily recognized by the close approximation of terminal hepatic vein and portal tracts (Wanless et al., 2000; Alastair et al., 2012). After establishment of cirrhotic nodules, some nodules have one or more than two terminal veins whereas others do not even have one. Massive necrosis usually occurs in cirrhotic nodules containing terminal veins inside. In those cirrhotic nodules without central veins, necrosis does not occur, at least not in the early stage of disease. Whether these cirrhotic nodules will also develop necrosis probably depends on whether the necrosis in neighboring nodules is capable of breaking through the surrounding septa.

## Liver biopsy in monitoring MHN

Liver biopsy is a useful tool in managing patients with ALF. Given that it is based on necrotic area of the whole liver, the classic MHN/SMHN definition is impossible to adopt undergo while a patient is alive and does not receive a liver transplant. More importantly, the clinical outcome of a patient with acute liver failure in either a previously healthy liver or in a cirrhotic liver does not correlate with the extent of necrosis (Hanau et al., 1995; Chenard-Neu et al., 1996). Detecting severe necrosis rather than clarifying the degree of necrosis may be more important for the prognosis of a patient with ALF. Boyer and Klatskin successfully used liver biopsy to discriminate patients with “subacute hepatic necrosis” (SHN) from those with “classic necrosis” according to whether or not the lesion displayed pan-lobular necrosis (Boyer and Klatskin, 1970). Biopsy identified SHN showed great prognostic value (Boyer and Klatskin, 1970). More recently, Dechene and colleagues used mini laparoscopy to perform liver biopsies in 39 patients with ALF (Dechêne et al., 2014). They showed that liver biopsy in patients with ALF and severe coagulopathy was safe. Further, detecting cell death and regeneration markers such as M30 and Ki67 with IHC staining may identify patients who may recover spontaneously or need a liver transplant. Thus, liver biopsy is a very useful tool for monitoring progression and prognosis of patients with acute decompensation. If possible, performing serial liver biopsies can provide dynamic information about disease progression and help clinicians pay special attention to those patients with MHN.

Besides liver biopsy, non-invasive marker(s), which can be used to predict outcome of ALF, are required for future routine clinical practice. However, finding useful and specific maker(s) for ALF patients is a big challenge. An ideal predictive marker should meet the following requirements: (1) The marker is closely associated with one or several core patho-physiological features of ALF, e.g., death of parenchymal cell, systemic or local inflammation, cell regeneration; (2) It reflects the severity of the disease; (3) It indicates the prognosis of ALF; and (4) It helps clinicians to make a decision on if and when the patient with ALF requires a liver transplantation. Given the complexity of human ALF, we still do not know most of the mechanisms by which liver cells are destroyed within short time, nor the mechanisms by which the liver can regenerate itself within a few hours (Samuel and Ichai, 2010). In addition, many factors, including etiology, predisposition of individuals (such as gender, age, genetics), hepatic, clinical, and biological status on admission and at the peak of deterioration and others, influence disease progression and final outcome of ALF. At the present time, no single parameter can reflect this complexity of ALF and is thus recognized as a routine clinical marker. Multiple prognostic criteria, such as the King's College Hospital (KCH) criteria (O'Grady et al., 1989), Clichy–Villejuif criteria (Bismuth et al., 1995) or Mayo end-stage liver disease score (MELD) (Malinchoc et al., 2000), remain the most widely used prognostic criteria for ALF. At the current stage, new molecular markers probably make best sense when incorporated into established prognostic models, for example, CK18-M65-based MELD (Bechmann et al., 2010).

## Summary

Massive hepatic necrosis in ALF and ACLF has unique pathophysiological characters including extremely rapid parenchymal cell death and dead cell removal. These features result in very high mortality in patients. On the other hand, they make it very difficult to study the underlying mechanisms. Due to the rapid and severe course of the disease, obtaining liver tissues undergoing MHN is almost impossible in clinical practice. Thus, though MHN has been recognized for more than one century, only preciously few studies with solid pathologic data are available (Lucke, 1944; Lucke and Mallory, 1946). The mechanisms underlying MHN are unknown. It seems obvious that the etiology underlying ALF or ACLF plays a key role in MHN and liver regeneration. Acetaminophen induced necrosis is more serious than that induced by non-acetaminophen toxins (Bechmann et al., 2010). So far, how etiology impacts MHN in ALF or ACLF is largely unanswered. In the future, clinical studies involving histology data will remain very limited. Establishing useful animal models will help hepatologists to understand the detailed mechanisms of MHN.

Like the occurrence of MHN, LPC activation is very rapid. However, in contrast to MHN, LPC-mediated liver regeneration can sustain until parenchymal mass is restored completely if the patient survives. Thus, clinical studies to investigate how LPCs replace lost parenchymal cells are feasible. Although recent pluripotent stem cell studies provide exciting results in establishing the “first example of a functional human organ from pluripotent stem cells” *in vitro* (Takebe et al., 2013), clinical application is not yet conceivable in the near future. One of the main reasons is that these engineered hepatocytes lack real bile canaliculi (Michalopoulos et al., 2013). Unlike these artificial hepatocytes, LPC-originated hepatocytes are fully functional with intact polarity, where one end maintains the link between hepatocyte canaliculi and the biliary tree while the other surfaces link to membranes of neighboring hepatocytes as the parenchyma fills in (Theise et al., 2013). Thus, uncovering mechanisms of LPC activation, proliferation and differentiation will also help us to identify useful pluripotent stem cells to treat patients with liver failure.

Acute-on-chronic liver failure is a recent recognized disease entity. So far, a consensus definition of ACLF has not been reached. Given that the disease occurs in a previously cirrhotic liver, it is not known whether different etiologies, e.g., cirrhotic livers due to HBV or alcoholic liver disease, will present similar histological changes. In contrast to ALF, no recognized animal models have been established. Mechanisms and pathophysiology of ACLF are largely obscure. These issues are awaiting further intensive investigation in the future.

**Figure 3:**
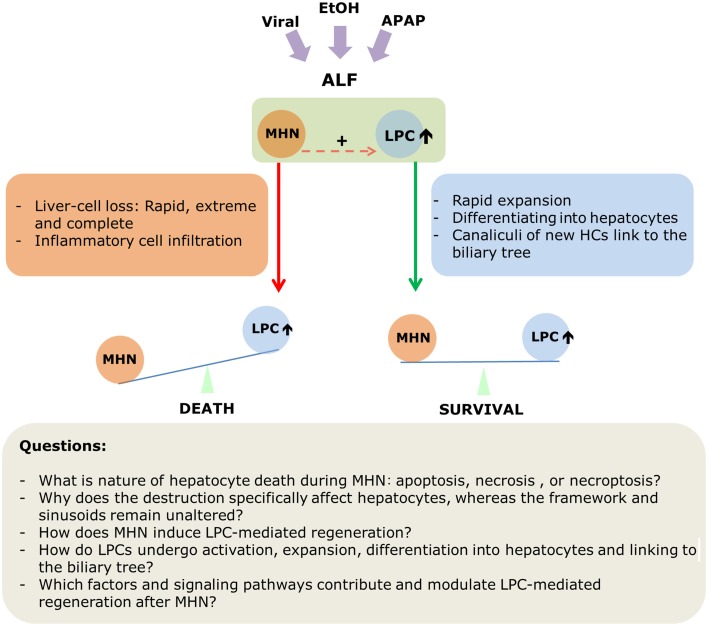
**A scheme depicting MHN and LPC-mediated regeneration in acute liver failure**.

The following should be emphasized at the end of this review: Massive hepatic necrosis and liver progenitor cell-mediated regeneration are not isolated events during acute liver failure that occurs either in healthy or in diseased liver. On the contrary, these two phenomena happen almost simultaneously. LPC-mediated regeneration is manifest at an extremely early phase of destruction (Lucke, 1944; Lucke and Mallory, 1946). On the one hand, MHN induces activation of LPCs and subsequent regeneration. On the other hand, whether LPCs can provide enough functional hepatocytes within a short time to restore liver mass and function determines destiny of patients who are suffering from ALF or ACLF. Thus, these two processes comprise the “two sides of one coin.” Figure 3 summarizes the current view of MHN and LPCs-mediated regeneration in ALF and the critical issues to be addressed in the future.

## Financial support

The study was supported by the Returned Overseas Chinese Scholars, State Education Ministry (Starting Research Foundation for the Returned Overseas Chinese Scholars, J20050337491010-G50523), People's Republic of China (HW), Else-Kröner Fresenius (HW, SD), the Chinese Nature Science Foundation, grant number: 30770962, 30971333, 81170421, 81470869, and Chinese High Technology “863”programme, grant number: 2006AA02A411 (HL); BMBF “Virtual liver” and “cell therapy” 01GN0987 (SD), Deutsche Forschungsgemeinschaft DO373/6-1, DO 373/8-1 (SD), TRR-SFB 77 (SD), IT-Liver ITN training grant (SD).

### Conflict of interest statement

The authors declare that the research was conducted in the absence of any commercial or financial relationships that could be construed as a potential conflict of interest.

## Abbreviations

ACLF: acute-on-chronic liver failure
ALF: acute liver failure
APACHE: acute physiology score, age points, chronic health points
HBcAg: hepatitis B core antigen
HBV: hepatitis B virus
CARS: compensation anti-inflammatory response syndrome
CDE: choline-deficient, ethionine-supplemented
CLIF-SOFA: chronic liver failure-sequential organ failure assessment score
DR: ductular reaction
ECM: extracellular matrix
INR: international normalized ratio
LPC: liver progenitor cells
MELD: model for end-stage liver disease
MHN: massive hepatic necrosis
MODS: multiple organ dysfunction syndrome
SHN: subacute hepatic necrosis
SIRS: systemic inflammatory response syndrome.
